# Prior-Assisted Hierarchical ADMM Decoding for Punctured Globally Coupled LDPC Codes

**DOI:** 10.3390/e28070815

**Published:** 2026-07-17

**Authors:** Wenbo Shi, Wenlong Xie, Jiashen Hu, Lishan Liu

**Affiliations:** 1 MoDo Institute of Technology, Henan University of Science and Technology, Luoyang 471023, China; 2 Longmen Laboratory, Luoyang 471000, China; xiewenlong@stu.haust.edu.cn (W.X.); hujiasen@stu.haust.edu.cn (J.H.); liulishan@stu.haust.edu.cn (L.L.); 3 School of Mathematics and Statistics, Henan University of Science and Technology, Luoyang 471023, China

**Keywords:** low-density parity-check (LDPC) codes, globally coupled LDPC (GC-LDPC), alternating direction method of multipliers (ADMMs), belief propagation (BP), hybrid decoding, parameter adaptation

## Abstract

Future wireless networks require channel coding schemes that can provide high reliability, low latency, and strong adaptability under finite-blocklength and structurally heterogeneous transmission scenarios. Globally coupled low-density parity-check (GC-LDPC) codes are promising for such systems because their coupled structure can enhance error-correction capability, but the additional global constraints also increase decoding complexity and make conventional fixed-parameter decoders less effective. This paper proposes a prior-assisted hierarchical alternating direction method of multipliers (ADMMs) decoding framework for GC-LDPC codes. The proposed decoder first partitions the GC-LDPC parity-check structure into two local subgraphs and performs tuned ADMM decoding on the local blocks in parallel. The local decoding outputs are then merged and verified by the full GC-LDPC parity-check matrix. If the merged local decision satisfies all global constraints, it is directly accepted, thereby avoiding unnecessary full-graph decoding. Otherwise, a global fallback ADMM decoder is activated. In this stage, the channel log-likelihood ratios are fused with soft priors extracted from the local ADMM outputs, where prior clipping and conflict scaling are introduced to control unreliable or contradictory local information. The resulting fused reliability information is used to guide full-matrix ADMM decoding. This local-to-global strategy reduces unnecessary global iterations while preserving the ability to enforce global consistency when local decoding is insufficient. Simulation-oriented metrics, including bit error rate, frame error rate, local pass rate, global fallback rate, global fallback success rate, and average iteration count, are used to evaluate reliability and decoding efficiency. The proposed framework provides an average-complexity-aware and reliability-aware decoding approach for advanced channel coding in future wireless networks.

## 1. Introduction

### 1.1. Background and Related Work

Low-density parity-check (LDPC) codes are a class of linear block codes defined by sparse parity-check matrices [1,2]. Due to their strong error-correction capability and implementation-friendly graph structure, LDPC codes have been widely studied and deployed in modern communication and storage systems [3,4,5]. In next-generation wireless networks, channel coding is expected to support high reliability, high spectral efficiency, low latency, and flexible operation under heterogeneous service requirements [6]. These requirements make the decoding problem more than an error-rate optimization task: the decoder must also control average iteration count, memory access, message-passing overhead, and robustness under finite-blocklength and punctured transmission settings.

Belief propagation (BP) and its variants remain among the most widely used LDPC decoding methods because they exploit the sparse Tanner graph through iterative exchange of variable-node and check-node messages [5,7]. Many recent studies continue to improve BP-type decoding by changing the message representation or scheduling strategy. For example, check-belief propagation reduces cumulative message computations by propagating check-node reliability information [8], while layered and residual-based scheduling schemes attempt to accelerate convergence for 5G LDPC codes by better ordering message updates [9]. These methods confirm that decoding complexity is strongly affected not only by the code structure but also by the way the Tanner graph is traversed. However, for large or structurally coupled codes, full-graph BP still requires repeated updates over all edges, and its performance can be affected by short cycles, message correlation, and high memory traffic.

Optimization-based decoding offers a complementary route. Linear programming (LP) decoding formulates maximum-likelihood decoding through a convex relaxation, and ADMM-based methods make this formulation more practical by splitting global constraints into local parity-polytope projection subproblems [10,11,12,13]. Recent ADMM-LDPC work has further targeted low-iteration regimes, multiplier update rules, and projection complexity. Representative examples include efficient ADMM decoding for limited iteration budgets [14], symmetric ADMM updates for penalized LP decoding [15], simplified or approximate check-polytope projection methods [16,17], and free-of-check-polytope-projection formulations [18]. These studies show that ADMM decoding is attractive when structured optimization, parallel local updates, and reliability-aware soft outputs are useful. Nevertheless, most ADMM improvements are developed for conventional LDPC matrices and do not directly address the local–global consistency problem in globally coupled LDPC codes.

Globally coupled LDPC (GC-LDPC) codes extend conventional LDPC constructions by connecting multiple local component codes through additional global constraints [19,20]. This coupling can improve finite-length error-rate performance and provide flexible structural design, but it also introduces heterogeneous degrees and long-range constraint interactions. Recent work has explored GC-LDPC and related coupled structures in several directions, including coded modulation [21], rate-compatible Matryoshka GC-LDPC codes [22], spatially and globally coupled LDPC codes for NAND Flash memories [23], hierarchical GC-LDPC codes with adaptive two-phase decoding [24], and variable-node-based tail-biting GC-LDPC codes [25]. These studies reinforce the value of coupling, but they also show that the decoder must exploit the induced structure carefully. In particular, local subcodes can often be processed in parallel, whereas global constraints are needed to remove residual inconsistency across local blocks.

### 1.2. Research Gap and Motivation

The natural decoding strategy for GC-LDPC codes is therefore not purely local and not purely global. A purely local decoder can reduce latency by processing component subgraphs independently, but local validity does not imply validity under the complete GC-LDPC parity-check matrix. A full-matrix decoder, on the other hand, enforces all local and global constraints, but it processes the entire graph even for frames that could have been resolved by local decoding and a simple global syndrome check. Recent two-phase GC-LDPC decoders have addressed this tension by combining local and global decoding stages, using mechanisms such as early termination, forced convergence, or adaptive activation of global checks [23,24,26]. These approaches demonstrate the importance of stage-aware decoding, but most of them are built around BP, Min-Sum, or related message-passing decoders rather than an ADMM framework that can reuse relaxed local optimization outputs as soft reliability information.

This distinction is important for punctured GC-LDPC decoding. In punctured positions, the channel LLRs carry no direct observation and are commonly initialized to zero. Decoding must therefore rely more heavily on parity-check constraints and on reliability information inferred from neighboring variables. If a local ADMM decoder has already produced relaxed outputs close to binary values, these outputs contain information about local convergence and bit confidence. Discarding this information when activating a full-matrix fallback decoder wastes useful computation; however, injecting it directly into the global decoder is also risky. Local estimates may be unreliable when a local block has not passed its own checks, and they may conflict with the original channel LLRs. Prior information in optimization-based decoding therefore needs to be bounded, reliability-aware, and conflict-sensitive, consistent with the broader idea that weighted or adaptive reliability information can improve decoding only when it is controlled properly [27,28].

These observations motivate a decoder that combines three ideas. First, it should exploit the local sub-block structure of the GC-LDPC parity-check matrix to avoid unnecessary full-graph iterations. Second, it should use the complete parity-check matrix as a verification and correction mechanism so that local decisions are accepted only when they satisfy all global coupling constraints. Third, when global fallback decoding is necessary, it should reuse the soft information generated by local ADMM decoders, but only after applying local-confidence gating, magnitude clipping, and channel-conflict scaling. The goal is not to replace global consistency with local confidence, but to guide full-matrix ADMM with controlled prior information while preserving the final authority of the complete GC-LDPC syndrome check.

### 1.3. Proposed Idea and Main Contributions

This paper proposes a prior-assisted hierarchical ADMM decoding framework for punctured GC-LDPC codes. The decoder first partitions the full parity-check matrix into two local subgraphs and performs tuned ADMM decoding on the two local blocks in parallel. Their hard decisions are concatenated into a full-length local candidate and verified using the complete GC-LDPC parity-check matrix. If the candidate satisfies all parity checks, it is accepted directly, avoiding unnecessary full-matrix ADMM iterations. Otherwise, the decoder activates a global fallback ADMM stage. In this stage, the original channel LLRs are fused with bounded pseudo-priors extracted from the relaxed local ADMM outputs. The prior contribution is attenuated when a local block is unreliable, clipped to avoid overconfidence, and reduced when it conflicts with the channel observation. The fused reliability vector is then used by the full-matrix ADMM decoder to enforce global consistency.

The novelty is therefore not only the use of two decoding stages, which has appeared in previous BP/Min-Sum-based GC-LDPC decoders, but the use of ADMM relaxed local outputs as controlled pseudo-prior information for a complete-matrix ADMM fallback decoder. The contributions are summarized as follows:

1. A two-phase ADMM decoder, denoted by TPD-ADMM, is developed for punctured GC-LDPC codes. Different from existing two-phase BP/Min-Sum decoders, both the local component decoders and the fallback decoder are formulated in the ADMM framework, and complete-matrix syndrome verification is used as the decision authority between direct local acceptance and global fallback.
2. A bounded pseudo-prior reuse mechanism is introduced for the global fallback stage. Instead of restarting full-matrix ADMM only from the channel LLRs, TPD-ADMM transfers relaxed local ADMM outputs into the fallback decoder through a fused LLR vector, which is especially useful for punctured positions whose channel LLRs contain no direct observations.
3. Reliability controls are designed for local-prior injection. Local-confidence gating, magnitude clipping, and channel-prior conflict scaling prevent unreliable or contradictory local information from dominating the complete-matrix ADMM decoder.
4. Complexity and latency accounting is provided for the proposed local/global ADMM structure. The analysis separates total arithmetic workload from the parallel local-stage latency proxy, and the simulations validate the resulting behavior through BER/FER, fallback statistics, average-iteration trends, and operation-count trends.

### 1.4. Organization of the Paper

The remainder of this paper is organized as follows. Section 2 introduces the system model and decoding preliminaries, including the LDPC representation, channel reliability model, ADMM decoding principle, BP baseline, and the structural characteristics of GC-LDPC codes. Section 3 presents the proposed prior-assisted hierarchical ADMM decoder, including local subgraph decoding, full-matrix verification, prior-controlled global fallback, final decision selection, offline parameter tuning, and complexity analysis. Section 4 reports the simulation framework and performance comparisons used to assess reliability and computational efficiency. Section 5 concludes the paper and discusses future research directions.

## 2. System Model and Preliminaries

### 2.1. LDPC Representation and Channel Reliability Model

Let an LDPC code be defined by a sparse parity-check matrix $H\in {\left\{0,1\right\}}^{m\times n}$, where *n* is the code length and *m* is the number of parity-check equations. A binary vector $x=(x_{1},x_{2},\ldots ,x_{n})$ is a valid codeword if

(1) $$Hx^{T}=0,$$

where the operation is performed over GF(2). The sparse matrix *H* can be represented by a Tanner graph. Let V={1,2,…,n} and C={1,2,…,m} denote the variable-node and check-node sets, respectively. The neighboring sets are defined as

(2) $$N\left(i\right)=\left\{j\in C|H_{j,i}=1\right\},\;M\left(j\right)=\left\{i\in V|H_{j,i}=1\right\}.$$

This paper considers punctured GC-LDPC transmission over a Gray-coded QPSK-AWGN channel. The encoder first generates a length-$N_{m}$ mother codeword, and a deterministic puncturing pattern P is then applied before transmission. Puncturing means that the coded bits indexed by P are omitted from the transmitted sequence after encoding; it is different from shortening because these bits are not fixed to known values and are not removed from the mother-code. The detailed construction of P is given in Section 2.4.

The receiver is described by the full-length channel log-likelihood ratio (LLR) vector $\gamma =(\gamma_{1},\gamma_{2},\ldots ,\gamma_{N_{m}})$. A positive $\gamma_{i}$ favors bit 0, while a negative $\gamma_{i}$ favors bit 1. For i∉P, $\gamma_{i}$ is computed from the received channel sample. For i∈P, no coded symbol is transmitted and no direct channel observation is available; therefore, the corresponding channel LLR is initialized to zero, i.e., $\gamma_{i}=0$. Thus, punctured positions remain unknown variable nodes with neutral channel evidence.

To avoid ambiguity between the mother-code rate and the rate after puncturing, let $N_{m}$ denote the original mother-code length, $N_{\mathrm{tx}}=N_{m}-P$ denote the number of transmitted coded bits, and *K* denote the number of information bits. The mother-code rate is $R_{m}=\frac{K}{N_{m}}$, whereas the effective transmitted rate after puncturing is $R_{\mathrm{eff}}=\frac{K}{N_{\mathrm{tx}}}=\frac{K}{N_{m}-P}$.

For the short GC-LDPC codebooks used in the simulations, $N_{m}=44Z$, K=20Z, and P=4Z, so $N_{\mathrm{tx}}=40Z$. Thus, $R_{m}=20/44$, while $R_{\mathrm{eff}}=20/40=1/2$. Throughout the simulation section, the reported rate 1/2 refers to the effective transmitted rate after puncturing rather than the mother-code rate.

Under equiprobable codewords, decoding can be viewed as selecting the codeword that best matches the received reliability information:

(3) $$\hat{x}=\arg\min_{x\in {\left\{0,1\right\}}^{n}}\gamma^{T}x,\;s.t.\;Hx^{T}=0.$$

This compact formulation is sufficient for the proposed decoder because the online algorithm only requires the full-length LLR vector, the puncturing rule, and the relevant local or global parity-check matrices.

### 2.2. ADMM Decoding Principle

ADMM-based LDPC decoding reformulates the parity-constrained decoding problem as a relaxed optimization problem. For each check node *j*, let $P_{j}x$ extract from *x* the variables connected to that check node, and let $z_{j}$ be a local replica constrained to the local parity polytope $P_{j}$. A standard relaxed ADMM decoding form is

(4) $$\min_{x,\{z_{j}\}}\;\gamma^{T}x,\;s.t.\;P_{j}x=z_{j},\;z_{j}\in P_{j},\;x\in {\left[0,1\right]}^{n},\;\forall j\in C.$$

The ADMM iterations alternate among three operations: updating the relaxed variable vector *x*, projecting each local replica $z_{j}$ onto its local parity polytope, and updating the corresponding dual variables. The iteration stops when the hard decision satisfies the parity-check equations or when the maximum number of iterations is reached. In this work, ADMM is used in two roles: local ADMM decodes the subgraphs first, and global ADMM is activated only when full-matrix verification fails.

In the punctured setting, ADMM is still applied to the full mother-code variable vector. The puncturing pattern has already been incorporated into the reliability vector γ: transmitted coordinates use channel LLRs, whereas punctured coordinates use $\gamma_{i}=0$. Therefore, the ADMM update equations are unchanged except for this reliability initialization, and the punctured variable nodes remain in the syndrome check.

More specifically, the ADMM decoder used in this work follows the standard ADMM-LP decoding framework of Liu and Draper [13]. Using the scaled dual variable $u_{j}$ and penalty parameter μ>0, the augmented update for the relaxed variable can be written as

(5) $$x^{\left(k+1\right)}=\arg\min_{0\leq x\leq 1}\left[\gamma^{T}x+\frac{\mu }{2}\sum_{j\in C}{∥P_{j}x-z_{j}^{\left(k\right)}+u_{j}^{\left(k\right)}∥}_{2}^{2}\right].$$

For a variable node *i* with degree $d_{i}$, this step gives the component-wise clipped update

(6) $$x_{i}^{\left(k+1\right)}=\Pi_{\left[0,1\right]}\left(\frac{\sum_{j\in N(i)}\left(z_{j,i}^{\left(k\right)}-u_{j,i}^{\left(k\right)}\right)-\gamma_{i}/\mu }{d_{i}}\right),$$

where $\Pi_{\left[0,1\right]}\left(\cdot \right)$ denotes scalar projection onto the interval [0,1]. After the variable update, each local replica is projected onto the corresponding parity polytope:

(7) $$z_{j}^{\left(k+1\right)}=\Pi_{P_{j}}\left(P_{j}x^{\left(k+1\right)}+u_{j}^{\left(k\right)}\right),\;j\in C.$$

The scaled dual variable is then updated by

(8) $$u_{j}^{\left(k+1\right)}=u_{j}^{\left(k\right)}+P_{j}x^{\left(k+1\right)}-z_{j}^{\left(k+1\right)},\;j\in C.$$

The hard decision is obtained by thresholding $x^{\left(k+1\right)}$ at 0.5 and checking the corresponding syndrome. In the proposed decoder, the local ADMM stages apply the same update principle to $H_{1}$ and $H_{2}$, whereas the fallback stage applies it to the complete matrix $H_{\mathrm{GC}}$. The local and global stages may use different tuned values of μ, iteration limits, and reliability-control parameters, but the underlying ADMM update structure remains the same.

The relaxed ADMM output *x* is also useful beyond its final hard decision. Since values close to 0 or 1 indicate stronger local confidence, the proposed global fallback stage converts local relaxed outputs into bounded soft priors. This role is especially important for punctured positions: compared with a non-punctured codeword, where each variable node has direct channel evidence, a punctured variable would otherwise enter the fallback decoder with neutral reliability. Reusing the locally inferred relaxed value therefore transfers constraint-derived reliability into the complete-matrix ADMM stage, while the clipping and confidence controls described later prevent this information from becoming an unbounded hard constraint.

### 2.3. Baseline BP Decoding

BP decoding is used as a representative message-passing baseline. It exchanges LLR messages between variable nodes and check nodes over the Tanner graph and updates posterior bit reliabilities iteratively. Decoding terminates when the hard decision satisfies all parity checks or when the iteration limit is reached. Although BP is effective for many LDPC codes, applying it directly to the complete GC-LDPC graph requires repeated full-graph message updates. Its computational order is commonly expressed as

(9) $$O(I_{\max}|E|),$$

where $I_{\max}$ is the maximum number of BP iterations and |E| is the number of Tanner-graph edges. This full-graph update pattern motivates a hierarchical strategy that avoids global processing when local decoding is already sufficient.

### 2.4. Puncturing Pattern and Circulant-Column Selection

The puncturing procedure used in this work is defined at the quasi-cyclic (QC) base-matrix column level. Let $H_{s}$, s∈{1,2}, denote one local component matrix. Each nonzero base-matrix entry of $H_{s}$ is expanded into a Z×Z circulant permutation matrix, and each base-matrix column corresponds to one group of *Z* lifted variable nodes. Therefore, puncturing is not performed by randomly deleting isolated coded bits. Instead, once a circulant column is selected, all *Z* variable nodes represented by that base column are punctured together.

Using zero-based local indexing within one local component, if the *j*th circulant column is selected for puncturing, the associated punctured variable-node set is

(10) $$P_{j}^{\mathrm{loc}}=\left\{jZ,jZ+1,\ldots ,jZ+Z-1\right\}.$$

If multiple circulant columns are selected, with selected column-index set $S_{p}$, the punctured coordinate set in one local component is

(11) $$P^{\mathrm{loc}}=\underset{j\in S_{p}}{⋃}P_{j}^{\mathrm{loc}}.$$

This notation makes the selection of the *Z* variable nodes explicit: the choice is made at the base-column level, and the lifting factor *Z* determines the number of transmitted-codeword coordinates omitted by each selected column.

For the two-local-component GC-LDPC code used here, each local component contains 22 lifted variable-column groups. The implemented puncturing rule uses

(12) $$S_{p}=\left\{0,1\right\},\;p_{c}=\left|S_{p}\right|=2,$$

in each local component. Using one-based global variable-node indices for the full mother-codeword, the complete puncturing set is

(13) $$P=\{\;b\left(22Z\right)+jZ+r+1|b\in \left\{0,1\right\},\;j\in S_{p},\;r=0,1,\ldots ,Z-1\;\}.$$

Thus, each local component has $p_{c}Z=2Z$ punctured variable nodes, and the complete GC-LDPC code has $P=2p_{c}Z=4Z$ punctured variable nodes. For the short codebooks in Table 1, this gives $N_{\mathrm{tx}}=N_{m}-P=40Z$ transmitted coded bits and the effective transmitted rate $R_{\mathrm{eff}}=1/2$.

The phrase “removal of columns” should be understood in this transmission sense. Let $T_{s}$ be the non-punctured coordinates of local component *s*, and let $S_{T_{s}}$ be the corresponding coordinate-selection matrix. The transmitted local codeword segment is

(14) $$x_{s,\mathrm{tx}}=S_{T_{s}}x_{s}.$$

Equivalently, for transmission-length accounting, one may view the transmitted part of $H_{s}$ as omitting the columns indexed by $P^{\mathrm{loc}}$. However, these columns are not deleted from the mother parity-check matrices used for decoding. The variable nodes corresponding to the punctured columns remain in $H_{1}$, $H_{2}$, and $H_{\mathrm{GC}}$ and continue to participate in the local and global parity checks; only their channel observations are absent.

The selected circulant columns are determined by four design factors. First, QC structure preservation requires puncturing complete lifted columns rather than isolated bits. Second, rate compatibility is controlled by changing $p_{c}$, the number of punctured circulant columns per local component. Third, local-component symmetry is maintained by using the same $p_{c}$ and the same selected base-column pattern in both local components, avoiding an artificial imbalance between the two local code rates. Fourth, connectivity and decoding stability are considered by retaining all punctured variable nodes in the mother Tanner graph and avoiding a puncturing rule that would remove the influence of the global coupling checks. The fixed choice $S_{p}=\left\{0,1\right\}$ is therefore a reproducible, QC-compatible puncturing pattern that gives the target transmitted rate while preserving the local-global constraint structure.

### 2.5. Block Structure and Decoding Challenges of the GC-LDPC Code

The GC-LDPC code considered in this work has a two-local-block structure. Its full parity-check matrix can be written as

(15) $$H_{\mathrm{GC}}=\left[\begin{array}{cc} H_{1} & 0\\ 0 & H_{2}\\ H_{g,1} & H_{g,2} \end{array}\right],$$

where $H_{1}$ and $H_{2}$ are the local parity-check matrices of the two component LDPC subcodes, and $H_{g,1}$ and $H_{g,2}$ form the global coupling checks.

In this paper, local decoding means ADMM decoding performed only on one diagonal component matrix, i.e., on $H_{1}$ or $H_{2}$ with the corresponding local LLR vector. It enforces the parity checks inside one component subcode and can be executed independently for the two local blocks. In contrast, global decoding means ADMM decoding performed on the complete matrix $H_{\mathrm{GC}}$, including the two local row groups and the coupling rows. The two concepts are linked by complete-matrix syndrome verification: local decoding first produces a merged candidate, and global decoding is activated only when that candidate does not satisfy the complete GC-LDPC parity-check matrix.

This block structure directly motivates the proposed hierarchical decoder. Since the two local row blocks are independent before the global rows are considered, the two local ADMM decoders can run separately and in parallel on $\left(H_{1},\gamma_{1}\right)$ and $\left(H_{2},\gamma_{2}\right)$. Their hard outputs can then be concatenated to form a full-length candidate, and the complete matrix $H_{\mathrm{GC}}$ is retained as the final verification matrix for the coupling checks.

The global rows are the main source of residual inconsistency after local decoding. They also identify the situation in which a full-matrix fallback decoder is necessary. The proposed method consequently uses local decoding as a lower-workload first stage, complete-matrix syndrome verification as a trigger, and prior-assisted global ADMM as a correction stage only when the concatenated local candidate is not globally consistent.

## 3. Proposed Prior-Assisted Hierarchical ADMM Decoding Framework

### 3.1. Design Rationale and Overall Flow

The proposed method is built around three design requirements implied by the GC-LDPC block structure. First, local decoding should be exploited because many constraints are sparse and confined within individual component blocks, but the resulting local decisions must still be verified by the complete GC-LDPC parity-check matrix. Second, full-matrix ADMM decoding should not be applied unconditionally, because frames that already satisfy all complete-matrix checks after local decoding do not require global iterations. Third, when global fallback is required, the relaxed local ADMM outputs should be reused as bounded pseudo-LLR prior information rather than as unbounded hard constraints. This prior information must be controlled by local reliability, clipping, and channel-conflict scaling so that unreliable local evidence does not dominate the original channel observations.

The complete online decoding flow is summarized in Algorithm 1.

### 3.2. Local Subgraph ADMM Decoding and Full-Matrix Verification

Following the block form in Section 2.5, the decoder uses a verification-triggered local-to-global flow. It first decodes the two diagonal local subgraphs and invokes full-matrix decoding only when the concatenated local decision fails to satisfy the complete parity-check matrix. Given the channel LLR vector γ, the local LLR vectors are extracted as

(16) $$\gamma_{1}=\gamma {{|}_{V_{1}},\;\gamma_{2}=\gamma |}_{V_{2}}.$$

The two local ADMM decoders operate independently:

(17) $$\left(x_{b}^{L},{\hat{x}}_{b}^{L}\right)=D_{\mathrm{ADMM}}\left(H_{b},\gamma_{b};\theta_{L,b}\right),\;b\in \left\{1,2\right\},$$

where $x_{b}^{L}$ is the soft decision output of local ADMM, ${\hat{x}}_{b}^{L}$ is its hard decision, and $\theta_{L,b}$ denotes the local ADMM parameter set for block *b*. Since $V_{1}$ and $V_{2}$ are disjoint, no local–local decision conflict exists during merging. The local hard decisions are concatenated into the full-length candidate

(18) $${\hat{x}}_{\mathrm{loc}}=\left[{\hat{x}}_{1}^{L},{\hat{x}}_{2}^{L}\right].$$

For later prior control, the local syndrome and the number of locally unsatisfied checks are defined as

(19) $$s_{b}=H_{b}{\left({\hat{x}}_{b}^{L}\right)}^{T},\;U_{b}\left({\hat{x}}_{b}^{L}\right)=\left|\left\{j\in C_{b}|{\left(s_{b}\right)}_{j}\neq 0\right\}\right|,\;b\in \left\{1,2\right\}.$$

The concatenated local candidate is then verified by the complete matrix:

(20) $$H_{\mathrm{GC}}{\hat{x}}_{\mathrm{loc}}^{T}=0.$$

If this condition holds, the decoder outputs ${\hat{x}}_{\mathrm{loc}}$ directly. This verification step prevents unnecessary full-matrix ADMM decoding for frames that have already been resolved by the local decoders and the global coupling checks. If the condition is not satisfied, the decoder enters the prior-assisted global fallback stage.
**Algorithm 1** Online TPD-ADMM decoding for punctured GC-LDPC codes**Input:** 
Received LLR vector γ; local matrices $H_{1},H_{2}$; complete matrix $H_{\mathrm{GC}}$; tuned ADMM and prior-control parameters.**Output:** 
Final hard decision $\hat{x}$. 1:Extract $\gamma_{1}$ and $\gamma_{2}$ from the full-length LLR vector γ. 2:Run the local ADMM decoders on $\left(H_{1},\gamma_{1}\right)$ and $\left(H_{2},\gamma_{2}\right)$ in parallel to obtain $x_{1}^{L},x_{2}^{L}$ and ${\hat{x}}_{1}^{L},{\hat{x}}_{2}^{L}$. 3:Concatenate ${\hat{x}}_{1}^{L}$ and ${\hat{x}}_{2}^{L}$ to form ${\hat{x}}_{\mathrm{loc}}$ and compute its syndrome using $H_{\mathrm{GC}}$. 4:**if** $H_{\mathrm{GC}}{\hat{x}}_{\mathrm{loc}}^{T}=0$ **then** 5:   Set $\hat{x}\leftarrow {\hat{x}}_{\mathrm{loc}}$. 6:   **return** $\hat{x}$. 7:**end if** 8:Map $x_{1}^{L}$ and $x_{2}^{L}$ to pseudo-LLR priors. 9:Apply local-confidence gating, magnitude clipping, and channel-conflict scaling.10:Fuse the controlled priors with γ and run the global ADMM fallback decoder on $H_{\mathrm{GC}}$.11:Select $\hat{x}$ by complete-matrix validity; if both candidates are invalid, apply the lexicographic syndrome-weight and channel-metric rule.12:**return** $\hat{x}$.

### 3.3. Prior-Controlled Global Fallback Decoding

When full-matrix verification fails, the relaxed local ADMM outputs are reused as bounded pseudo-LLR prior information. Because the two local variable sets are disjoint, each variable obtains its pseudo-prior from exactly one local decoder. For variable node $i\in V_{b}$, the local pseudo-LLR prior is generated as

(21) $$p_{i}=\alpha_{b}\eta_{b}\left(1-2x_{b,i}^{L}\right),\;i\in V_{b},$$

where $\eta_{b}\geq 0$ is the prior gain for local block *b* and $\alpha_{b}\in \left[0,1\right]$ is a block-level local confidence factor. The term $\left(1-2x_{b,i}^{L}\right)$ is obtained from the usual binary-to-sign reliability convention: bit value 0 corresponds to positive LLR evidence and bit value 1 corresponds to negative LLR evidence. Because the ADMM output $x_{b,i}^{L}\in \left[0,1\right]$ is relaxed rather than strictly binary, the affine mapping converts values close to 0 into positive pseudo-LLR evidence, values close to 1 into negative pseudo-LLR evidence, and values near 0.5 into weak evidence. Thus, $p_{i}$ is not treated as a true posterior LLR derived from a probability ratio; it is a calibrated pseudo-prior used to guide the global fallback decoder, consistent with the use of controlled reliability weights in optimization-based and reweighted LP/ADMM decoding [27,28].

**Figure 1:**
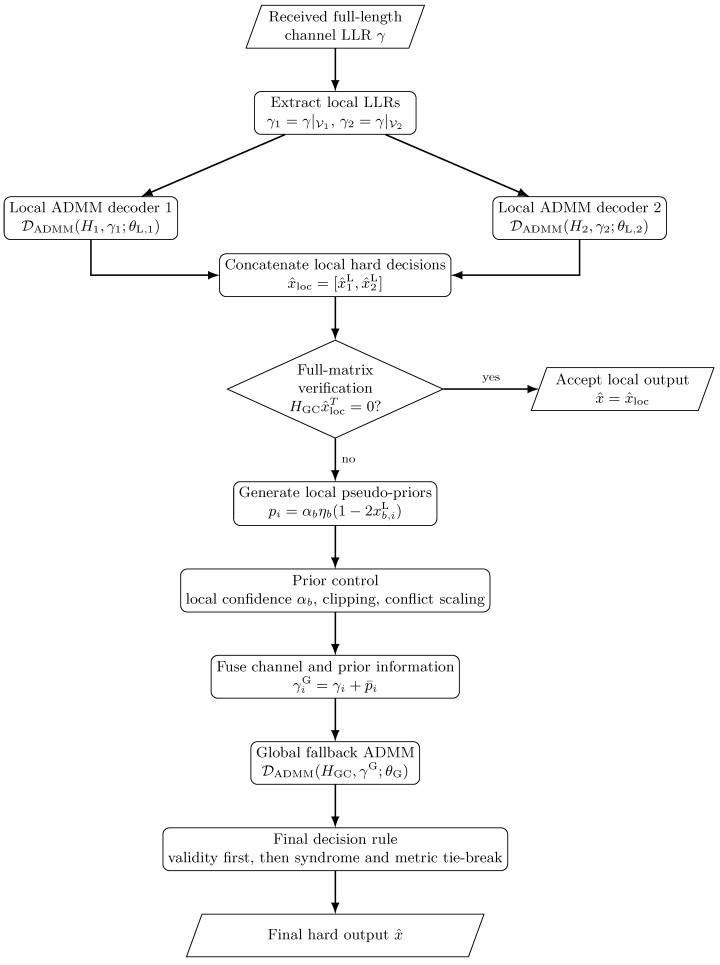
Online flowchart of the proposed prior-assisted hierarchical ADMM decoder. The complete GC-LDPC syndrome check determines whether the local candidate can be accepted directly or whether controlled pseudo-prior information from the local ADMM outputs should be injected into the global fallback ADMM decoder.

The online prior-assisted fallback procedure is illustrated in Figure 1.

The scale of this pseudo-prior is deliberately bounded. The gain $\eta_{b}$ determines how strongly the relaxed local output is allowed to modify the channel LLR, while the subsequent clipping threshold $p_{\max}$ prevents any local output from becoming an effectively hard constraint. The attenuation factor $\alpha_{\mathrm{fail}}$ reduces the contribution of a local block whose hard decision fails its own parity checks, and the conflict-scaling factor β further suppresses pseudo-priors whose signs disagree with the channel observation. Therefore, the global decoder receives reliability guidance from the local ADMM stage, but the injected prior remains controlled by explicit magnitude, validity, and channel-consistency tests.

A simple local-validity gate is used to prevent unreliable local blocks from being over-emphasized:

(22) $$\alpha_{b}=\left\{\begin{array}{cc} 1, & U_{b}\left({\hat{x}}_{b}^{L}\right)=0,\\ \alpha_{\mathrm{fail}}, & U_{b}\left({\hat{x}}_{b}^{L}\right)>0, \end{array}\right.\;0\leq \alpha_{\mathrm{fail}}<1.$$

Therefore, a local block that satisfies its own parity checks contributes its full pseudo-prior strength, whereas a local block that fails its own checks contributes only an attenuated pseudo-prior. This block-level control complements the bit-level controls below. To avoid overconfident pseudo-priors, the prior magnitude is clipped:

The binary local-confidence gate is used because local syndrome validity is deterministic, inexpensive to compute, and directly tied to whether the local hard decision satisfies the local parity constraints. More refined confidence measures, such as the distance of the relaxed components from 0.5, primal and dual ADMM residual norms, or the magnitude of the local syndrome weight, can provide additional reliability information. However, they introduce extra thresholds and may make the online decoder more dependent on manually selected confidence parameters. In this work, the binary gate is therefore used as the simplest reproducible block-level reliability test, while clipping and channel-conflict scaling provide finer bit-level protection against unreliable local priors.

(23) $${\tilde{p}}_{i}=\max\left(-p_{\max},\min\left(p_{i},p_{\max}\right)\right),\;p_{\max}>0.$$

If the clipped pseudo-prior conflicts with the channel LLR, its magnitude is further reduced:

(24) $${\bar{p}}_{i}=\left\{\begin{array}{cc} \beta {\tilde{p}}_{i}, & \gamma_{i}{\tilde{p}}_{i}<0,\\ {\tilde{p}}_{i}, & \gamma_{i}{\tilde{p}}_{i}\geq 0, \end{array}\right.$$

where 0≤β≤1 is the conflict-scaling factor. For punctured positions, the channel LLR is zero by construction, and the pseudo-prior is therefore controlled by the local confidence gate and clipping rather than by channel-conflict scaling. The fused LLR for global fallback decoding is

(25) $$\gamma_{i}^{G}=\gamma_{i}+{\bar{p}}_{i},\;i=1,2,\ldots ,n.$$

The global ADMM fallback decoder then operates on the complete parity-check matrix:

(26) $$\left(x_{G},{\hat{x}}_{G}\right)=D_{\mathrm{ADMM}}\left(H_{\mathrm{GC}},\gamma^{G};\theta_{G}\right),$$

where $\theta_{G}$ denotes the parameter set used by the full-matrix global ADMM decoder.

### 3.4. Final Decision Rule

The final decision rule considers three cases. If the concatenated local candidate already satisfies the complete GC-LDPC parity-check matrix, it is selected directly. If local verification fails but the global fallback output satisfies the complete matrix, the global fallback output is selected. If both candidates are invalid, the decoder compares their complete-matrix syndrome weights and then uses the channel metric as a tie breaker.

For any binary hard decision *x*, define the number of unsatisfied complete-matrix checks as

(27) $$U\left(x\right)=\left|\left\{j\in C_{\mathrm{GC}}|{\left(H_{\mathrm{GC}}x^{T}\right)}_{j}\neq 0\right\}\right|,$$

and define the channel metric as

(28) $$M\left(x\right)=\gamma^{T}x.$$

The final hard output is then

(29) $$\hat{x}=\left\{\begin{array}{cc} {\hat{x}}_{\mathrm{loc}},\; & H_{\mathrm{GC}}{\hat{x}}_{\mathrm{loc}}^{T}=0,\\ {\hat{x}}_{G},\; & H_{\mathrm{GC}}{\hat{x}}_{\mathrm{loc}}^{T}\neq 0\;\mathrm{and}\;H_{\mathrm{GC}}{\hat{x}}_{G}^{T}=0,\\ \arg\min_{y\in \{{\hat{x}}_{\mathrm{loc}},{\hat{x}}_{G}\}}^{\mathrm{lex}}\left(U(y),M(y)\right),\; & \mathrm{otherwise}. \end{array}\right.$$

The notation $\arg\min^{\mathrm{lex}}$ denotes lexicographic minimization. In the last case, the candidate with fewer unsatisfied complete-matrix checks is preferred first, and if the two candidates have the same number of unsatisfied checks, the one with the smaller channel metric is selected. When both candidates are invalid, the frame is still counted as a decoding failure in FER evaluation; the last branch only provides a deterministic hard output and may reduce BER.

### 3.5. Offline Parameter Tuning

The local and global ADMM stages may use different iteration limits, penalty parameters, relaxation factors, continuation schedules, and degree-dependent parameter tables. These parameters interact with the pseudo-prior gains, clipping threshold, local-confidence attenuation, and conflict-scaling factor. Therefore, differential evolution (DE) is used offline to tune the parameter vector

(30) $$\theta =\left[\theta_{L},\theta_{G},\theta_{\deg},\theta_{\mathrm{prior}}\right],$$

where $\theta_{L}$ and $\theta_{G}$ contain the local and global ADMM parameters, $\theta_{\deg}$ contains degree-dependent table entries, and $\theta_{\mathrm{prior}}$ contains the pseudo-prior control parameters such as $\eta_{1}$, $\eta_{2}$, $p_{\max}$, $\alpha_{\mathrm{fail}}$, and β. For a representative SNR set S, the tuning objective is written as

(31) $$\theta^{★}=\arg\min_{\theta \in \Theta }J\left(\theta \right),$$

with the normalized objective

(32) $$\begin{array}{cc} J\left(\theta \right)=\sum_{\mathrm{snr}\in S}( & w_{\mathrm{BER}}\frac{{\mathrm{BER}}_{\mathrm{snr}}\left(\theta \right)}{{\mathrm{BER}}_{\mathrm{snr}}^{\mathrm{ref}}}+w_{\mathrm{FER}}\frac{{\mathrm{FER}}_{\mathrm{snr}}\left(\theta \right)}{{\mathrm{FER}}_{\mathrm{snr}}^{\mathrm{ref}}}+w_{\mathrm{iter}}\frac{{\bar{K}}_{\mathrm{snr}}\left(\theta \right)}{K_{\max}}+w_{\mathrm{fb}}P_{\mathrm{fb},\mathrm{snr}}\left(\theta \right)), \end{array}$$

where ${\bar{K}}_{\mathrm{snr}}\left(\theta \right)$ is the average iteration count, $P_{\mathrm{fb},\mathrm{snr}}\left(\theta \right)$ is the global fallback rate, and the positive reference values ${\mathrm{BER}}_{\mathrm{snr}}^{\mathrm{ref}}$ and ${\mathrm{FER}}_{\mathrm{snr}}^{\mathrm{ref}}$ are baseline or design-normalization constants. This normalization prevents the iteration-count term from dominating the error-rate terms only because of scale differences. DE is performed before deployment; online decoding only loads the tuned parameter set.

Table 2 summarizes the pseudo-prior control parameters, their offline search ranges, and the representative tuned values used in the reported tuning run. In the implementation, the valid-block and invalid-block prior strengths are specified directly as pass and fail gains; this is equivalent to using $\eta_{b}$ for locally valid blocks and $\alpha_{\mathrm{fail}}\eta_{b}$ for locally invalid blocks. The ranges are finite and deliberately conservative: the pass gain is allowed to vary from no prior injection to a moderate pseudo-LLR gain, the fail gain remains small unless locally invalid blocks are useful, $p_{\max}$ limits the maximum injected magnitude, and β is restricted to an attenuation factor. In the simulations, the tuned values are selected for each representative codebook and SNR operating region before decoding, and the online decoder only reads the resulting parameter table. No DE search is performed during online decoding.

The sensitivity of the decoder to these parameters is mainly governed by the bounded-prior design. If $\eta_{b}$ is too small, the global fallback approaches ordinary channel-only ADMM decoding and the benefit of local-output reuse decreases. If $\eta_{b}$ is too large, incorrect local information may dominate the channel evidence, but this effect is limited by $p_{\max}$, $\alpha_{\mathrm{fail}}$, and β. Similarly, a smaller β makes the decoder more conservative when the local pseudo-prior conflicts with the channel LLR, whereas a larger β trusts the local ADMM output more strongly. This tradeoff explains why the parameters are tuned offline for representative operating regions rather than fixed universally across all codebooks and SNR values.

For reproducibility, Table 3 reports the representative DE settings used to obtain the tuned parameter set. The listed settings are taken from the historical tuning log used for the paper results, while the parameter bounds and objective terms follow the tuning script. In the implemented objective, the BER term is compared with the BP reference BER at the same SNR through the ADMM/BP BER ratio. The fitness used in the tuning script is

(33) $$\begin{array}{cc} J_{\mathrm{DE}}\left(\theta \right)=\sum_{\mathrm{snr}\in S}( & 1000\max\left\{\log_{10}r_{\mathrm{BER}},0\right\}+200\;\mathrm{FER}+100\left(1-P_{\mathrm{valid}}\right)\\ \; & +0.01\;\bar{K}-50\min\left\{-\log_{10}r_{\mathrm{BER}},3\right\}), \end{array}$$

where $r_{\mathrm{BER}}={\mathrm{BER}}_{\mathrm{ADMM}}/{\mathrm{BER}}_{\mathrm{BP}}$ at the same SNR, $P_{\mathrm{valid}}$ is the final complete-matrix valid rate, and $\bar{K}$ is the average total iteration count. Thus, candidates are penalized when their BER is worse than BP, rewarded in a bounded way when their BER is better than BP, and also evaluated by FER, final validity, and average iteration count.

### 3.6. Complexity and Latency Analysis

#### 3.6.1. Computational Workload Model

Let $K_{1}$ and $K_{2}$ be the local ADMM iteration numbers, and let $K_{G}$ be the global fallback iteration number. Let $|E_{1}|$ and $|E_{2}|$ denote the edge numbers of the two local graphs, let $|E_{g}|$ denote the number of edges in the global coupling part, and let $|E_{\mathrm{GC}}|$ denote the number of edges in the complete GC-LDPC graph. The numerical edge counts below correspond to the representative Z80G1T2 configuration used in the iteration-count and operation-count analysis: $|E_{1}\left|=\right|E_{2}|=2680$, the global coupling part contains $|E_{g}|=800$ edges, and the complete graph contains $|E_{\mathrm{GC}}|=6160$ edges. For other lifting factors with the same base-matrix structure, these counts scale approximately linearly with *Z*; equivalently, $|E_{q}\left(Z\right)|=\left(Z/80\right)|E_{q}\left(80\right)|$ for q∈{1,2,g,GC}. Therefore, the fixed edge numbers should be interpreted as the Z80G1T2 representative case rather than universal constants for all codebooks. If $C_{\Pi ,1}$, $C_{\Pi ,2}$, and $C_{\Pi ,\mathrm{GC}}$ denote the corresponding parity-polytope projection costs, the local-stage workload is

(34) $$C_{\mathrm{local}}=O\left(K_{1}\left(\right|E_{1}|+C_{\Pi ,1})+K_{2}\left(\right|E_{2}\left|+C_{\Pi ,2}\right)\right).$$

This expression measures the total arithmetic workload of the two local decoders. If the two local ADMM decoders are executed in parallel, the local-stage latency is dominated by the larger of the two local decoding costs rather than by their sum. The projection costs depend on the parity-polytope projection routine and the check-node degrees.

Every frame also requires complete-matrix syndrome verification after local decoding. This verification has cost

(35) $$C_{\mathrm{ver}}=O(|E_{\mathrm{GC}}\left|\right).$$

For frames that require fallback, the additional global ADMM cost is

(36) $$C_{\mathrm{global}}=O\left(K_{G}\left(\right|E_{\mathrm{GC}}\left|+C_{\Pi ,\mathrm{GC}}\right)\right),$$

and constructing the controlled pseudo-prior vector has cost

(37) $$C_{\mathrm{prior}}=O\left(n\right).$$

If the global fallback rate is $P_{\mathrm{fb}}$, the average online complexity is approximated as

(38) $$E\left[C\right]\approx C_{\mathrm{local}}+C_{\mathrm{ver}}+P_{\mathrm{fb}}\left(C_{\mathrm{global}}+C_{\mathrm{prior}}\right).$$

Thus, the online decoder avoids complete-matrix ADMM iterations for frames that pass local decoding and full-matrix verification, while retaining a global correction path for difficult frames. The additional verification and prior-construction costs are non-iterative and are usually smaller than the full-matrix ADMM iteration cost.

#### 3.6.2. Decoding Latency

The computational complexity above measures the total arithmetic workload, whereas decoding latency is determined by the sequential critical path from receiving the full LLR vector to producing the final hard decision. This distinction is important for the proposed decoder because the two local ADMM decoders can be executed in parallel. Therefore, the local-stage workload is the sum of two local workloads, but the local-stage latency is governed by the slower local branch.

Let $T_{\mathrm{split}}$ denote the time for extracting local LLR vectors, $T_{\mathrm{ver}}$ the complete-matrix syndrome verification time, $T_{\mathrm{prior}}$ the time for controlled pseudo-prior construction, and $T_{\mathrm{sel}}$ the final decision-selection time. Let $1_{\mathrm{fb}}$ be the fallback indicator, where $1_{\mathrm{fb}}=1$ if the locally merged candidate fails the complete-matrix parity check and $1_{\mathrm{fb}}=0$ otherwise. The online latency of one decoded frame can be expressed as

(39) $$T_{\mathrm{TPD}}=T_{\mathrm{split}}+T_{\mathrm{local}}+T_{\mathrm{ver}}+1_{\mathrm{fb}}\left(T_{\mathrm{prior}}+T_{\mathrm{global}}\right)+T_{\mathrm{sel}},$$

where TPD-ADMM denotes the proposed two-phase hierarchical decoder. If the two local decoders are implemented in parallel,

(40) $$T_{\mathrm{local}}=\max\left(T_{L,1},T_{L,2}\right),\;T_{L,b}=K_{b}T_{\mathrm{iter},b}^{L},\;b\in \left\{1,2\right\}.$$

Here, $T_{\mathrm{iter},b}^{L}$ is the latency of one local ADMM iteration on $H_{b}$. A serial implementation gives the conservative upper bound $T_{\mathrm{local}}=T_{L,1}+T_{L,2}$. For the fallback stage,

(41) $$T_{\mathrm{global}}=K_{G}T_{\mathrm{iter}}^{G},$$

where $T_{\mathrm{iter}}^{G}$ is the latency of one ADMM iteration over the complete matrix $H_{\mathrm{GC}}$. The prior-construction latency is non-iterative and scales linearly with the code length because it consists of pseudo-LLR mapping, local-confidence attenuation, clipping, channel-conflict scaling, and LLR fusion:

(42) $$T_{\mathrm{prior}}=O\left(n\right).$$

Let

(43) $$P_{\mathrm{fb}}=\mathrm{Pr}\left(H_{\mathrm{GC}}{\hat{x}}_{\mathrm{loc}}^{T}\neq 0\right)$$

denote the global fallback rate. The average decoding latency is then approximated by

(44) $$\begin{array}{cc} E[T_{\mathrm{TPD}}]\approx & T_{\mathrm{split}}+E\left[\max\left(K_{1}T_{\mathrm{iter},1}^{L},K_{2}T_{\mathrm{iter},2}^{L}\right)\right]+T_{\mathrm{ver}}+T_{\mathrm{sel}}\\ \; & +P_{\mathrm{fb}}\left(E\left[T_{\mathrm{prior}}|\mathrm{fb}\right]+E\left[K_{G}|\mathrm{fb}\right]T_{\mathrm{iter}}^{G}\right). \end{array}$$

For comparison, the average latency of direct full-matrix ADMM, denoted by FSD-ADMM, can be written as

(45) $$E\left[T_{\mathrm{FSD}}\right]\approx E\left[K_{\mathrm{FSD}}\right]T_{\mathrm{iter}}^{G}+T_{\mathrm{syn}}\left(H_{\mathrm{GC}}\right),$$

where $K_{\mathrm{FSD}}$ is the iteration count of the direct full-matrix ADMM decoder and $T_{\mathrm{syn}}\left(H_{\mathrm{GC}}\right)$ is the final syndrome-check time. Similarly, the full-graph BP baseline has the latency form

(46) $$E\left[T_{\mathrm{BP}}\right]\approx E\left[K_{\mathrm{BP}}\right]T_{\mathrm{iter}}^{\mathrm{BP}},$$

where $T_{\mathrm{iter}}^{\mathrm{BP}}$ includes the check-node update, variable-node update, and hard-decision verification in one BP iteration.

The proposed decoder has three latency regimes. In the best case, the locally merged candidate passes the complete-matrix verification, and the latency is

(47) $$T_{\min}=T_{\mathrm{split}}+T_{\mathrm{local}}+T_{\mathrm{ver}}+T_{\mathrm{sel}}.$$

In the worst case, both local decoders reach their maximum local iteration budgets and the global fallback decoder also reaches its maximum iteration budget:

(48) $$T_{\max}=T_{\mathrm{split}}+\max\left(K_{1,\max}T_{\mathrm{iter},1}^{L},K_{2,\max}T_{\mathrm{iter},2}^{L}\right)+T_{\mathrm{ver}}+T_{\mathrm{prior}}+K_{G,\max}T_{\mathrm{iter}}^{G}+T_{\mathrm{sel}}.$$

Therefore, the proposed hierarchical decoder does not unconditionally reduce the worst-case latency, because difficult frames may still require full-matrix ADMM after the local stage. Its latency advantage is mainly an average-latency advantage. When the SNR is low, $P_{\mathrm{fb}}$ is usually large and the fallback iterations dominate the critical path. As the SNR increases, direct acceptance may become more frequent and the fallback stage may also require fewer iterations because the local soft outputs are more reliable. The relative contribution of these two effects depends on the code structure and operating configuration. The sensitivity of average latency to the fallback rate is approximately

(49) $$\frac{\partial E[T_{\mathrm{TPD}}]}{\partial P_{\mathrm{fb}}}\approx E\left[T_{\mathrm{prior}}|\mathrm{fb}\right]+E\left[K_{G}|\mathrm{fb}\right]T_{\mathrm{iter}}^{G},$$

which shows that reducing the fallback rate and reducing the number of fallback iterations are the two main sources of latency reduction.

## 4. Simulation Results

This section presents the decoding results of the punctured GC-LDPC code over the Gray-coded QPSK-AWGN channel. Four representative operating configurations, denoted by Z80G1T2, Z80G4T2, Z160G1T2, and Z160G4T2, are used to examine reliability and decoding behavior under different parameter settings. The BP curves correspond to the full-graph BP baseline. The proposed prior-assisted hierarchical ADMM decoder is denoted by TPD-ADMM, and the direct full-matrix ADMM baseline is denoted by FSD-ADMM. These names are used consistently in the following performance, iteration-count, and operation-count discussions.

### 4.1. Error-Rate Performance

**Figure 2:**
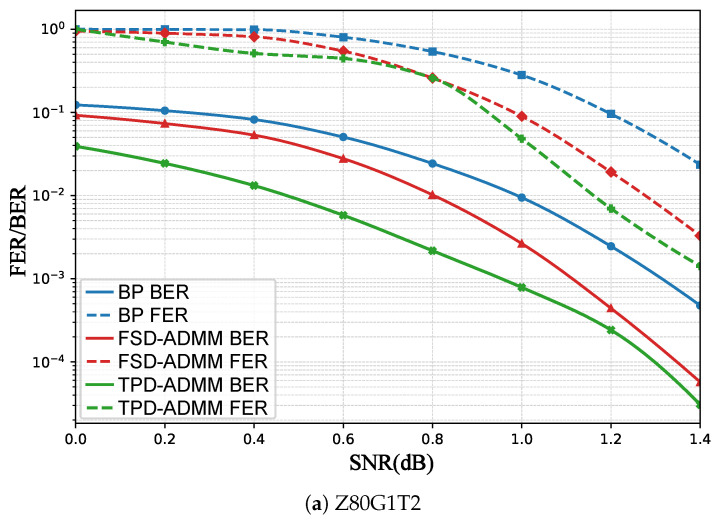

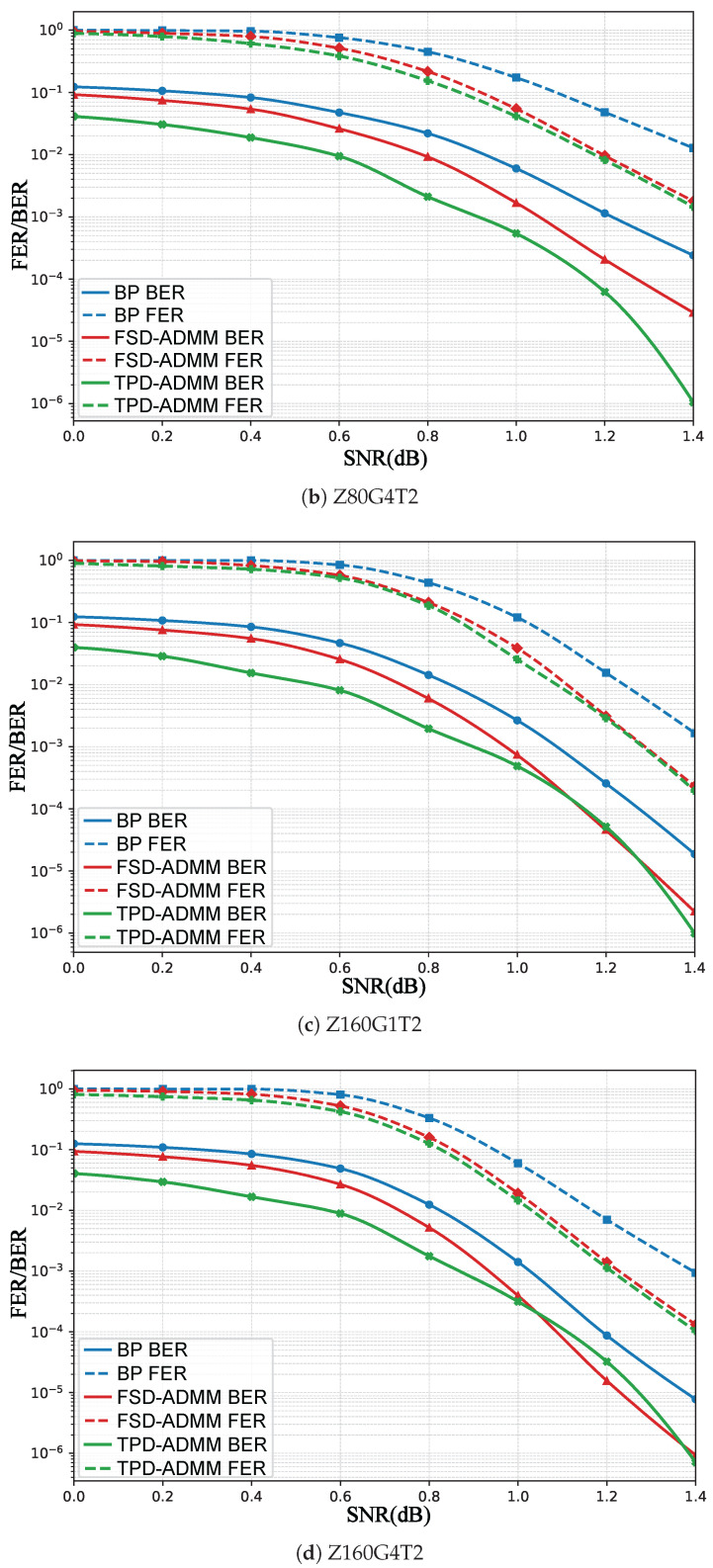
BER and FER comparisons of full-graph BP, FSD-ADMM, and TPD-ADMM under four representative configurations: (**a**) Z80G1T2, (**b**) Z80G4T2, (**c**) Z160G1T2, and (**d**) Z160G4T2. Here, FSD-ADMM is the direct full-matrix ADMM baseline, and TPD-ADMM is the proposed prior-assisted hierarchical ADMM decoder.

Figure 2 shows that TPD-ADMM achieves the best overall BER and FER performance among the three compared algorithms for all four tested configurations. FSD-ADMM consistently outperforms BP, which confirms the benefit of optimization-based decoding for the considered punctured GC-LDPC structure. More importantly, TPD-ADMM provides an additional gain over FSD-ADMM, especially in the medium- and high-SNR regions, which indicates that the local-to-global strategy and the injected local priors help the global stage converge to more reliable decisions.

The relative behavior of the four subfigures is also consistent with the expected effect of parameter refinement. Among the tested settings, Z80G1T2 is the weakest configuration, while Z160G4T2 provides the strongest error-rate performance. The gap between the proposed decoder and direct ADMM is particularly visible in FER, which suggests that the hierarchical decoder is more effective at suppressing residual frame-level failures after local decoding than either direct full-matrix ADMM or BP alone.

### 4.2. Local-to-Global Decoding Behavior

**Figure 3:**
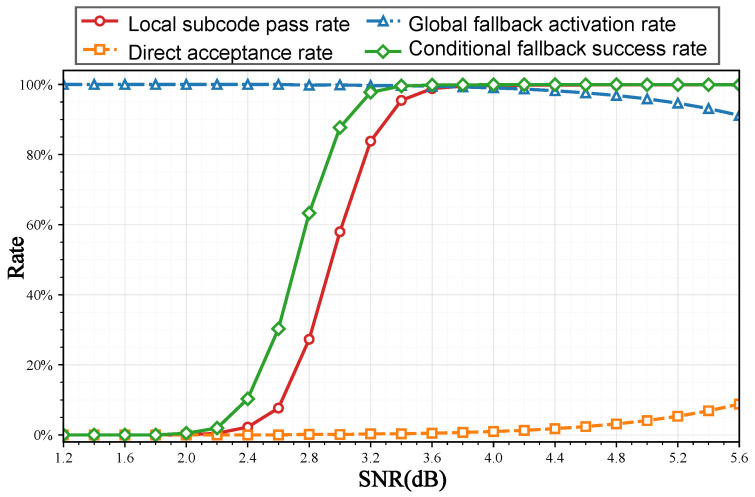
Local-to-global decoding behavior of the proposed TPD-ADMM decoder for the Z80G1T2 configuration. The local subcode pass rate measures whether the local ADMM stage satisfies the local parity checks, the direct acceptance rate measures whether the merged local candidate passes the complete GC-LDPC syndrome check, the global fallback activation rate measures the fraction of frames entering full-matrix fallback ADMM, and the conditional fallback success rate measures the success probability after fallback is activated.

Figure 3 further explains the internal behavior of TPD-ADMM. At a low SNR, the local subcode pass rate and the conditional fallback success rate are both low, so nearly all frames enter the global fallback stage and successful fallback correction is rare. As SNR increases, the local subcode pass rate rises rapidly, indicating that local ADMM produces increasingly reliable local decisions and soft outputs. The direct acceptance rate remains much lower than the local subcode pass rate, so complete-matrix verification is necessary and the global fallback decoder remains important for enforcing the coupling constraints. The increasing conditional fallback success rate also confirms that the controlled local priors become more useful in the fallback stage as local reliability improves.

### 4.3. Average Iteration Count

**Figure 4:**
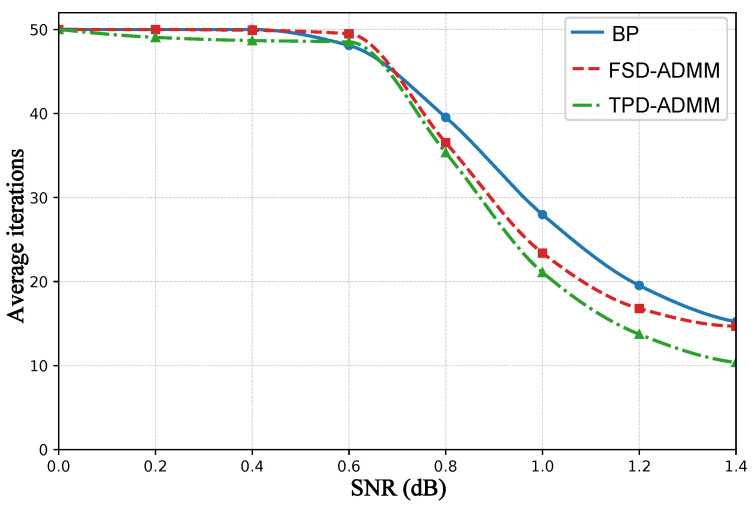
Average iteration count versus SNR for the Z80G1T2 configuration. Here, FSD-ADMM denotes the direct full-matrix ADMM baseline and TPD-ADMM denotes the proposed two-phase hierarchical decoder.

Figure 4 compares the average iteration counts of BP, FSD-ADMM, and TPD-ADMM. At low SNR, all three decoders operate close to the iteration limit, which reflects the greater decoding difficulty in that regime. As the SNR increases, the average iteration count decreases for all methods, but the reduction is fastest for TPD-ADMM. In the moderate-to-high SNR region, the proposed decoder requires fewer iterations than both direct ADMM and BP. This behavior is consistent with the intended design: the local phase produces more reliable soft outputs and a small but increasing fraction of directly accepted frames, while the frames entering fallback can benefit from controlled local priors that help the global ADMM phase converge with fewer iterations.

### 4.4. Latency Performance

The latency behavior follows the analytical model in Section 3.6. Since the actual wall-clock latency depends on the hardware platform, degree of local parallelism, memory bandwidth, and projection implementation, the simulation results are interpreted through the latency-related proxies reported in this work, namely the average iteration count and the operation-count trend. Under a parallel local implementation, the first-phase latency of TPD-ADMM is determined by the slower local ADMM branch rather than by the sum of the two local workloads. The additional full-matrix syndrome verification and prior-construction steps are non-iterative, whereas the global fallback ADMM phase is iterative and therefore dominates the extra latency whenever fallback is triggered.

At low SNR, the local candidate is less likely to satisfy the complete GC-LDPC parity-check matrix, so the fallback rate is high and the latency advantage of TPD-ADMM is limited. In this regime, many frames still require global ADMM iterations after the local phase, and the worst-case latency can approach the local-phase latency plus a full global decoding pass. In the moderate-to-high SNR region, however, the local pass probability increases and direct acceptance becomes more likely. For the tested local-to-global behavior in Figure 3, most frames still enter fallback, so the latency and iteration-count advantage of TPD-ADMM should be interpreted as coming from both early direct acceptance and, more importantly for this configuration, the ability of local soft priors to guide global ADMM and reduce unnecessary fallback iterations.

**Figure 5:**
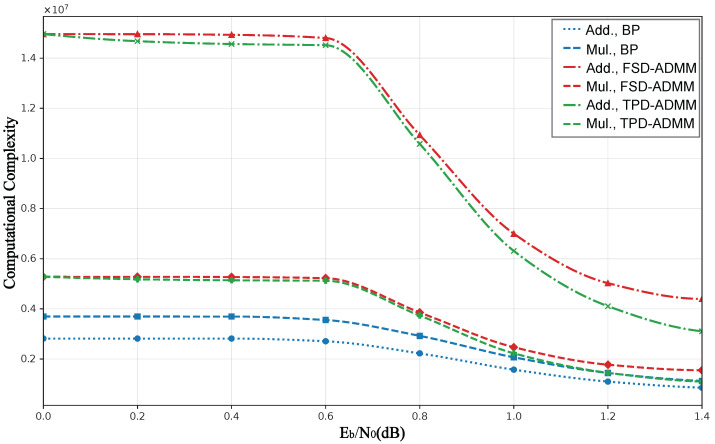
Average numbers of additions and multiplications versus $E_{b}/N_{0}$ for the Z80G1T2 configuration. Blue, red, and green curves denote BP, FSD-ADMM, and TPD-ADMM, respectively. For BP, circles on dotted curves and squares on dashed curves denote additions and multiplications, respectively. For FSD-ADMM, triangles on dash-dotted curves and diamonds on dashed curves denote additions and multiplications, respectively. For TPD-ADMM, crosses on dash-dotted curves and squares on dashed curves denote additions and multiplications, respectively.

The average-iteration result in Figure 4 therefore provides a latency-oriented indication: the faster decrease in the TPD-ADMM iteration curve means that fewer iterative update rounds remain on the online critical path as SNR increases. The operation-count result in Figure 5 further supports this interpretation. Since additions and multiplications are directly related to per-iteration processing time, the consistently lower operation count of TPD-ADMM indicates a smaller average processing burden per decoded frame. Thus, the proposed decoder mainly reduces average online latency rather than worst-case latency. Its latency gain is expected to be most visible in the SNR region where the fallback probability is low or where the prior-assisted global fallback requires fewer iterations than direct full-matrix ADMM.

### 4.5. Operation-Count Results

The computational trend in Figure 5 is consistent with the iteration behavior. At low SNR, both ADMM-based decoders incur large operation counts because many frames approach the maximum iteration budget. When the SNR increases, the average numbers of additions and multiplications decrease rapidly. The proposed TPD-ADMM remains consistently below FSD-ADMM in both addition and multiplication counts, showing that the local-to-global decoding flow reduces the average online workload. The complexity gap is particularly pronounced for additions, while the multiplication count of TPD-ADMM becomes close to that of BP at higher SNR. This result supports the complexity analysis in Section 3.6 and shows that the proposed decoder improves reliability without paying the full computational price of direct full-matrix ADMM on every frame.

This behavior can be understood from the workload decomposition in Section 3.6. FSD-ADMM applies ADMM iterations directly to the complete GC-LDPC matrix, so its dominant arithmetic term scales with the number of full-matrix iterations and the complete graph size, namely $K_{\mathrm{FSD}}\left(\right|E_{\mathrm{GC}}\left|+C_{\Pi ,\mathrm{GC}}\right)$. In contrast, TPD-ADMM first spends a fixed local decoding cost on the two smaller component graphs and then performs a complete-matrix syndrome verification. The expensive full-matrix ADMM update is invoked only through the fallback path. Therefore, the average cost is controlled not only by the local iteration numbers but also by the fallback activation probability and the number of global fallback iterations. The verification, prior construction, clipping, conflict scaling, and LLR fusion steps are non-iterative operations, so their costs increase only linearly with the code length and do not grow with the ADMM iteration budget.

The separate addition and multiplication curves also provide useful implementation insight. Additions are heavily used in variable-node accumulation, check-related aggregation, dual-variable updates, syndrome evaluation, and LLR fusion. As a result, reducing the number of full-matrix ADMM iterations directly lowers the addition count. Multiplications are mainly associated with penalty-parameter scaling, reliability weighting, prior-gain scaling, and conflict attenuation. These operations are still required in the proposed decoder, but many of them are applied on local blocks or only when fallback is activated. This explains why TPD-ADMM reduces both operation types, while the reduction in additions is more visible than the reduction in multiplications.

It should also be emphasized that the complexity advantage is an average-complexity advantage rather than an unconditional worst-case guarantee. In difficult low-SNR frames, TPD-ADMM may execute both local ADMM decoding and global fallback ADMM decoding, so the per-frame workload can approach the sum of local processing and one full-matrix fallback pass. However, in the moderate-to-high SNR region, the local outputs become more reliable and the fallback stage either becomes less frequent or converges with fewer global iterations. This is the operating region where the proposed local-to-global structure converts the GC-LDPC block structure into a measurable reduction in arithmetic workload.

## 5. Conclusions and Outlook

This paper proposed a prior-assisted hierarchical ADMM decoder for punctured GC-LDPC codes. The decoder exploited the local-block structure of the parity-check matrix by first performing independent ADMM decoding on the two component subgraphs, merging their hard decisions, and verifying the merged candidate with the complete GC-LDPC parity-check matrix. When the complete syndrome check failed, a full-matrix ADMM fallback decoder was activated, and bounded pseudo-priors extracted from the relaxed local ADMM outputs were fused with the original channel LLRs through local-confidence gating, magnitude clipping, and channel-conflict scaling.

The simulation results showed that TPD-ADMM improved BER and FER performance compared with the considered full-graph BP and FSD-ADMM baselines under the tested configurations. The local-to-global behavior analysis also showed that complete-matrix verification was necessary before accepting a locally decoded candidate. From the efficiency perspective, the average-iteration and operation-count results indicated that TPD-ADMM reduced average online effort in the moderate-to-high SNR region by combining occasional direct local acceptance with faster prior-assisted fallback convergence.

The advantage should be interpreted as an average-complexity and average-latency advantage rather than a worst-case guarantee because difficult frames may still require both local decoding and full-matrix fallback decoding. The current evaluation was based on simulation-oriented metrics rather than measured hardware runtime, so memory access, projection implementation, parallel scheduling, and fixed-point quantization effects remain open. Future work will focus on platform-based latency and throughput measurement, adaptive online tuning of prior-control parameters, larger GC-LDPC configurations, fixed-point implementation, and theoretical analysis of the convergence and failure behavior of prior-assisted ADMM decoding.

## Figures and Tables

**Table 1:** Parameters and puncturing settings of the short punctured GC-LDPC codebooks.

Codebook	*Z*	*G*	*T*	$p_{c}$	$M_{b}\times N_{b}$	$N_{m}=44Z$	$N_{\mathrm{tx}}=40Z$	M=24Z	K=20Z	P=4Z	$R_{m}$	$R_{\mathrm{eff}}$
Z80G1T2	80	1	2	2	24×44	3520	3200	1920	1600	320	20/44	1/2
Z80G4T2	80	4	2	2	24×44	3520	3200	1920	1600	320	20/44	1/2
Z160G1T2	160	1	2	2	24×44	7040	6400	3840	3200	640	20/44	1/2
Z160G4T2	160	4	2	2	24×44	7040	6400	3840	3200	640	20/44	1/2

**Table 2:** Pseudo-prior control parameters used in offline tuning.

Parameter	Search Range	Representative Tuned Value	Online Role
$\eta_{\mathrm{pass}}$	[0,5]	3.055182	Prior gain for locally valid blocks
$\eta_{\mathrm{fail}}=\alpha_{\mathrm{fail}}\eta_{b}$	[0,1.5]	0.009863	Prior gain for locally invalid blocks
$p_{\max}$	[0,3]	2.126492	Magnitude clipping threshold
β	[0,1]	0.454778	Scaling for channel-conflicting priors

**Table 3:** Representative DE tuning settings used for the prior-assisted hierarchical ADMM decoder.

Item	Value
SNR set S	{0.0,0.2,0.4,…,2.0} dB
BP BER reference	Measured BP BER at the same SNR
Population size	24 candidates in the Z80G1T2 tuning run
Evaluated generations	Complete generations 0–10; incomplete generations 11–12 not used
Mutation factor *F*	0.5
Crossover rate $C_{R}$	0.7
Evaluation stopping rule	200 frame errors or 2000 frames per SNR point
Representative candidate	g010_i012, fitness 3371.573
Online use	Load tuned parameters only; no online DE search

## Data Availability

The data supporting the findings of this study are available within the article. The source code and additional simulation data are available from the corresponding author upon reasonable request.

## Abbreviations

The following abbreviations are used in this manuscript:

ADMM	Alternating direction method of multipliers.
AWGN	Additive white Gaussian noise.
BER	Bit error rate.
BP	Belief propagation.
DE	Differential evolution.
FER	Frame error rate.
GC-LDPC	Globally coupled low-density parity-check.
LDPC	Low-density parity-check.
LLR	Log-likelihood ratio.
SNR	Signal-to-noise ratio.
FSD	Full-matrix decoding.
TPD	Two-phase decoding.
